# General and special education teachers’ readiness for artificial intelligence in classrooms: A structural equation modeling study of knowledge, attitudes, and practices in select UAE public and private schools

**DOI:** 10.1371/journal.pone.0331941

**Published:** 2025-09-12

**Authors:** Mohammad Fteiha, Mohammad Al-Rashaida, Mohammed Ghazal

**Affiliations:** 1 Department of Education, College of Arts and Sciences, Abu Dhabi University, Abu Dhabi, United Arab Emirates; 2 Department of Special and Gifted Education, College of Education, United Arab Emirates University, Al Ain, United Arab Emirates; 3 Department of Electrical and Computer Engineering, Abu Dhabi University, Abu Dhabi, United Arab Emirates; University of Science and Technology of Fujairah, YEMEN

## Abstract

As artificial intelligence (AI) reshapes global education systems, understanding educators’ readiness to integrate AI into classroom practices is essential. This study examines the knowledge, attitudes, and practices (KAP) of general and special education teachers in the United Arab Emirates (UAE) regarding AI in education. Drawing on the Concerns-Based Adoption Model (CBAM) and Universal Design for Learning (UDL), we used structural equation modeling (SEM) to assess the relationships among KAP domains, including the moderating effects of demographic factors such as teaching experience, academic role, and prior exposure to AI tools. Data were collected from 161 educators in selected public and private schools across four UAE emirates, with the majority representing private and urban school settings. The findings revealed that teachers’ attitudes significantly predicted AI-related classroom practices, whereas knowledge had a weaker, but positive association. Mediation analysis further showed that knowledge had a significant indirect effect on practice through attitudes, confirming the hypothesized KAP pathway. Moderation analyses highlighted the variability in AI engagement based on gender and academic position, suggesting differentiated readiness across the subgroups. This study contributes to global conversations on teacher preparedness by offering a model for assessing institutional and pedagogical readiness for AI integration in urban school contexts. Implications for professional development, inclusive curriculum design, and educational technology policy are discussed, with relevance to digitally transforming educational systems in comparable settings.

## Introduction

The rapid advancement of Artificial Intelligence (AI) is reshaping global education systems, compelling educators to acquire new competencies for effective AI integration into the classroom [1]. Governments increasingly recognize AI as central to innovation-driven reform, with education identified as a strategic domain for its deployment [2,3]. In the United Arab Emirates (UAE), National AI Strategy 2031 outlines an ambitious vision for embedding AI across sectors, including K–12 education, through digital competency development and smart learning environments [4]. Despite substantial infrastructure investments, AI implementation in classrooms remains uneven, particularly among general and special education teachers, owing to limited preparation and targeted training [5–8]. This highlights the pressing need to examine teachers’ readiness in terms of knowledge, attitudes, and classroom practices, which are pivotal for closing the gap between national policy ambitions and practical integration.

The challenges of the UAE reflect a broader global pattern. While 61% of countries acknowledge AI’s strategic importance in education, fewer than 30% have implemented comprehensive teacher training programs [1]. Similarly, the Organization for Economic Co-operation and Development [9] notes that although enthusiasm for AI is high, classroom-level integration remains constrained by limited teacher preparation, ethical uncertainties, and fragmented policy guidance. These global limitations resonate with the context of the UAE, where sociocultural diversity, varied school types, and uneven infrastructure shape how AI is adopted across educational settings [10–12]. The gap is even more pronounced in special education, where AI’s promise, for example, in personalized learning or assistive technologies, requires not only technical knowledge, but also ethical literacy and pedagogical adaptability [8,13]. These complexities underscore the need to holistically assess teacher readiness, accounting for diverse roles, contexts, and levels of support.

A growing body of research highlights the importance of assessing teacher readiness through cognitive, affective, and behavioral dimensions [14,15]. The knowledge, attitudes, and practices (KAP) model has emerged as a useful lens for analyzing these components in educational innovation, particularly in the context of AI [16,17]. While frameworks such as technological pedagogical content knowledge (TPACK) emphasize the integration of technology into pedagogical and content domains [18], the KAP model captures broader readiness by linking foundational knowledge, value-driven attitudes, and applied classroom practice [19].This distinction is particularly relevant for emerging technologies such as AI, where ethical perceptions and confidence often shape classroom use as much as technical skills [14,20].

Although prior research in UAE higher education settings generally indicates favorable KAP profiles [21,22], the K–12 landscape remains underexplored. In particular, there is a limited understanding of how general and special education teachers vary across these dimensions, which is essential for building inclusive and context-sensitive AI implementation strategies. In this study, *knowledge* refers to teachers’ understanding of AI functions and applications, *attitudes* represent their affective dispositions and beliefs about the role of AI in education, and *practice* encompasses their reported or intended instructional use of AI technologies. These definitions align with the study’s theoretical framework and guide instrument development and analysis. Recent scholarship emphasizes that teacher readiness spanning cognitive (knowledge), affective (attitudes), and behavioral (practice) domains remains a pivotal yet under-examined factor shaping the successful adoption of AI in school settings [14,15,23].

Despite the growing global interest in AI in education, few studies have systematically examined how teachers’ knowledge, attitudes, and practices interact in real-world school settings, especially in the Arab Gulf context. Most available research focuses on university faculty or relies on descriptive accounts without exploring the predictive relationships between KAP dimensions [17]. Furthermore, limited attention has been paid to how demographic variables such as gender, school type, teaching experience, and prior AI training may shape K–12 teachers’ readiness to implement AI [20,24,25].

Special education settings remain underrepresented in empirical studies on AI in education despite their unique potential to benefit from AI-enhanced personalization, accessibility, and inclusion. Recent reviews and case studies highlight how AI tools, such as adaptive platforms, speech recognition, and assistive robotics, can support learners with disabilities, yet teacher preparedness in these settings is still nascent and uneven [8,19,26–28].

Given the increasing policy emphasis on AI integration in education and the persistent gaps in teacher preparedness, this study aimed to examine the knowledge, attitudes, and practices of K–12 teachers in the UAE, with specific attention to both general and special education contexts. It also explores how demographic variables, including gender, school type, teaching experience, and prior AI training, shape teachers’ readiness to adopt AI. Building on the KAP framework, this study further investigated the extent to which knowledge and attitudes predict AI-related practices and whether these relationships are moderated by demographic factors. This study makes three key contributions: (1) it offers the first comprehensive empirical analysis of AI-related KAP profiles among UAE school teachers; (2) it integrates special education into mainstream AI readiness research; and (3) it provides evidence to guide inclusive, policy-aligned professional development initiatives that support equitable AI adoption in diverse educational settings. Drawing on the integrated theoretical framework, this study was guided by the following research questions (RQs) and hypotheses (H).

1. What are the perceived levels of knowledge, attitudes, and AI-related practices among general and special education teachers in the UAE?
2. Do demographic variables (e.g., gender, school type, teaching experience, academic position, and prior AI training) significantly influence teachers’ knowledge, attitudes, and practices toward AI?
3. To what extent do teachers’ knowledge and attitudes predict their AI-related practices in education?
4. Do demographic variables moderate the relationship between teachers’ knowledge or attitudes and their AI-related practices?

H1: Teachers’ knowledge of AI significantly and positively predicts their AI-related practices in education.

H2: Teachers’ attitudes toward AI significantly and positively predict their AI-related practices in education.

## Theoretical framework

The current study is grounded in a multi-theoretical framework that integrates psychological, pedagogical, and technological perspectives to explain AI adoption among K–12 educators. It combines the KAP model, Technology Acceptance Model (TAM), Self-Efficacy Theory, and the Diffusion of Innovations (DOI), along with principles from digital competence and inclusive education. Together, these frameworks offer a multidimensional explanation of how teachers’ cognitive, affective, and behavioral factors influence AI integration in schools, while accounting for contextual moderators.

## KAP model.

The KAP model, originally rooted in public health [29], assumes a sequential path from knowledge acquisition to attitudinal change to behavioral action. In the context of AI in education, teachers’ understanding of AI concepts (e.g., machine learning, automation, and data ethics) influences their affective responses, which in turn drive classroom implementation [14,15,20]. Recent studies have confirmed that enhanced AI knowledge improves both teacher attitudes and readiness to adopt AI tools [15,16,30]. KAP has been successfully adapted to educational innovation contexts in which ethical, technical, and pedagogical dimensions are interlinked [17,31].

### TAM.

The technology acceptance model [32] provides a cognitive lens for adoption behavior by emphasizing perceived usefulness and ease of use as precursors to technology acceptance. In AI education, these perceptions directly influence teachers’ attitudes and intentions [7,25,33]. For example, teachers are more likely to adopt AI when they believe it enhances instruction (performance expectancy) and is manageable within daily classroom routines (effort expectancy). Studies have shown that perceived usefulness enhances the positive influence of AI-related knowledge on teachers’ attitudes, whereas low perceived ease of use can inhibit behavioral intentions despite high awareness or training [28,33–35].

### Self-efficacy theory.

According to Bandura’s Social Cognitive Theory [36], self-efficacy reflects an individual’s belief in their ability to perform specific actions. In educational technology, teacher self-efficacy has consistently predicted willingness to experiment with digital tools, including AI applications [20,37]. Teachers with high AI self-efficacy are more likely to develop positive attitudes and engage in classroom implementation [25,37,38]. Moreover, self-efficacy mediates the link between knowledge and practice, particularly when ethical risks or task ambiguity are present [8,28,33].

### DOI.

DOI Theory [39] contextualizes how new technologies spread through social systems based on characteristics such as relative advantage, compatibility, complexity, and trialability. In the UAE and other MENA settings, factors such as leadership support, policy clarity, and peer influence have been shown to shape the diffusion of AI into education [11,40]. Teachers are more inclined to adopt AI if they align with existing pedagogical practices, reduce their workload, and are promoted by the school leadership [10,41].

### Digital Competency and Inclusive Education Models.

This framework incorporates a Digital Competence Framework for Educators (DigCompEdu) to address the technical and ethical readiness required for equitable AI use [42]. This model emphasizes teacher competencies in digital content creation, ethical data use, and critical evaluation of emerging tools [10,24]. Moreover, the Universal Design for Learning (UDL) principles provides a lens to assess how AI technologies can enhance personalization, flexibility, and equity, particularly in special education [8,13,26]. These models emphasize that inclusive and ethical AI integration must be supported by targeted training, professional development, and institutional infrastructure [23,25,27,33].

### Conceptual Model.

**Fig 1:**
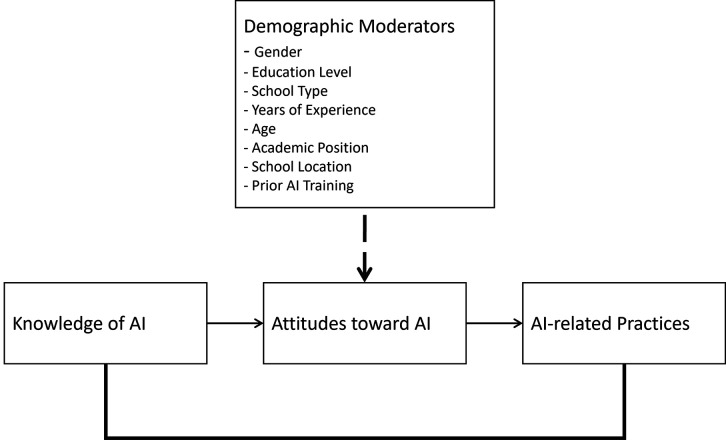
Conceptual framework guiding the study. Teachers’ knowledge of AI is hypothesized to directly influence their attitudes toward AI and their AI-related practices. Attitudes are proposed to mediate the relationship between knowledge and practice. In addition, demographic factors including gender, education level, years of experience, academic position, school location, school type, and prior AI training are hypothesized to moderate these relationships.

The hypothesized model positions teachers’ knowledge and attitudes as key predictors of AI-related practices, consistent with the KAP framework. It specifies both direct effects from knowledge to practice and from attitudes to practice as well as an indirect effect where attitudes mediate the relationship between knowledge and practice. The model also includes the moderating effects of demographic variables (e.g., gender, experience, prior AI training, academic position, and school type) on these relationships. This structure guides the study’s use of structural equation modeling (SEM) and moderation analysis to test the direct, mediated, and conditional effects outlined in the theoretical framework, as depicted in Fig 1.

## Method

This study employed a quantitative cross-sectional survey design to examine teachers’ knowledge, attitudes, and practices toward AI in education, along with the moderating effects of demographic factors.

### Participants

Participants in this study were in-service teachers employed full-time in public or private K–12 schools across the UAE, a federation of seven emirates (Abu Dhabi, Ajman, Dubai, Fujairah, Sharjah, Ras Al Khaimah, and Umm Al Quwain), with an estimated population of 10.48 million. As of the 2023–2024 academic year, the UAE has approximately 507 public and 403 private schools distributed across these emirates [43].

To be eligible, participants were required to (1) be full-time educators, (2) have at least one year of teaching experience, and (3) be directly involved in general or special education classroom instruction. Part-time and non-instructional staff members were excluded from the study. All responses were screened for eligibility prior to the analysis. Participants were recruited using a non-probability convenience sampling strategy selected for its pragmatic suitability in capturing early insights into emerging pedagogical trends among geographically dispersed educators in the UAE [44].

A total of 161 in-service K–12 educators participated in the study (see Table 1), comprising 127 females (79%) and 34 males (21%), from public and private schools located in four emirates: Abu Dhabi, Dubai, Sharjah, and Ajman. The sample was predominantly mid- to late-career professionals, with 85 teachers (53%) reporting more than 10 years of experience, and 76 (47%) aged between 31 and 40 years. The majority held bachelor’s degrees (n = 101, 63%), while 60 (37%) had postgraduate qualifications, reflecting the national emphasis on professional development. The roles were evenly split, with 85 general education teachers (53%) and 76 special education teachers (47%).

**Table 1:** Demographic characteristics of participants (N = 161).

Variable	n (%)
**Gender**	
Male	34 (21%)
Female	127 (79%)
**Educational Level**	
Bachelor	101 (63%)
Postgraduate	60 (37%)
**Type of School**	
Private	127 (79%)
Public	34 (21%)
**Years of Experience**	
1–3	29 (18%)
4–6	25 (15%)
7–10	22 (14%)
More than 10	85 (53%)
**Age Group**	
31–40	75 (47%)
41–50	60 (37%)
51–60	26 (16%)
**Academic Position**	
Special education teacher	76 (47%)
General education teacher	85 (53%)
**School location**	
Abu Dhabi	59 (37%)
Dubai	52 (32%)
Sharjah	30 (19%)
Ajman	20 (12%)
**Prior AI Training**	
**Yes**	77 (48%)
**No**	84 (52%)

Most participants were employed in private schools (n = 127, 79%), a pattern consistent with the UAE’s dominance in education in the private sector. In terms of location, the responses were concentrated in more urbanized emirates, with 59 participants (37%) from Abu Dhabi and 52 (32%) from Dubai. The sample comprised teachers from four of the seven emirates (Abu Dhabi, Dubai, Sharjah, and Ajman), with the majority (79%) employed in private schools and 21% in public schools.

The overall sample composition mirrors national workforce trends, particularly the high representation of experienced female educators [43]. The final sample size (n = 161) exceeded the recommended 5–10 participants per estimated parameter for structural equation modeling [45], supporting model stability and estimation accuracy.

#### Instrumentation.

This study employed a two-part survey. The first part collected demographic information, including gender, age group, academic position, school type, years of teaching experience, and school location, which were selected based on a literature review [3,46–51] and suggestions from experts who reviewed the research instrument.

The second part consisted of a newly developed 28-item instrument designed to assess in-service teachers’ engagement with AI technologies in K–12 education settings. The instrument was constructed to measure three latent constructs: teachers’ knowledge of AI, attitudes toward AI in education, and AI-related classroom practices, collectively referred to as the KAP-AIEd instrument. Item generation was guided by a theoretical framework and comprehensive review of the literature on AI in education [3,46–52]. The items were designed to capture multiple dimensions, including perceived benefits, ethical concerns, and practical applications of AI in education. Attitudinal and knowledge-related items were rated on a 5-point Likert scale (1 = Strongly Disagree to 5 = Strongly Agree), while practice-related items were measured on a 5-point frequency scale (1 = Not in Use to 5 = Fully or Mostly Implemented).

#### Content validity.

The content validity of the instrument was established through an expert review following the recommended best practices for scale development [53]. Three educational technology specialists affiliated with UAE institutions and one international specialist reviewed the draft questionnaire for conceptual alignment, cultural relevance, and linguistic clarity. Based on their feedback, three items (At10, K10, Pract8) were removed due to irrelevance to the instrument (e.g., At10: “AI can replace teachers”) This resulted in a 25-item instrument. Minor wording refinements were made to retain the items, and all revisions were incorporated prior to data collection.

#### Pilot testing.

After expert validation, a pilot test was conducted with eight postgraduate students specializing in education and educational technology. Participants assessed their clarity, comprehensibility, and flow. No items were removed, and only minor phrasing adjustments were made (e.g., simplified jargon in K7).

#### Exploratory factor analysis.

Following content validation and pilot testing, exploratory factor analysis (EFA) was conducted on the 25-item scale to examine the dimensionality of the *Knowledge, Attitudes, and Practices toward AI in Education* (KAP-AIEd) instrument, and to inform item refinement prior to confirmatory factor analysis (CFA). Principal axis factoring with varimax rotation was used to extract underlying factors*.*

Sampling adequacy was confirmed with a Kaiser–Meyer–Olkin (KMO) value of 0.861, which exceeds the recommended minimum of 0.60 [54]. Bartlett’s Test of Sphericity was also significant, χ²(300) = 3510.50, p < .001, indicating that the correlation matrix was factorable.

Three factors with eigenvalues greater than one were extracted, collectively explaining 67.84% of the total variance (Table 2). Factor I (Attitudes toward AI Integration; nine items) accounted for 25.38% of the variance, Factor II (Knowledge and Awareness of AI; nine items) explained 23.50%, and Factor III (AI-related Pedagogical Practice; seven items) accounted for 18.95%. All 25 items demonstrated loadings >0.40 and were retained for CFA [55].

**Table 2:** Summary of exploratory factor analysis.

Item	Factor I	Factor II	Factor III
At1	.873		
At2	.875		
At3	.907		
At4	.877		
At5	.874		
At6	.870		
At7	.777		
At8	.810		
At9	.497		
K1		.840	
K2		.841	
K3		.832	
K4		.891	
K5		.874	
K6		.901	
K7		.818	
K8		.497	
K9		.742	
Prac1			.847
Prac2			.842
Prac3			.799
Prac4			.854
Prac5			.855
Prac6			.729
Prac7			.718

*Note.* Factor I = Attitudes toward AI Integration; Factor II = Knowledge and Awareness of AI; Factor III = AI-related Pedagogical Practice

#### Confirmatory factor analysis.

**Fig 2:**
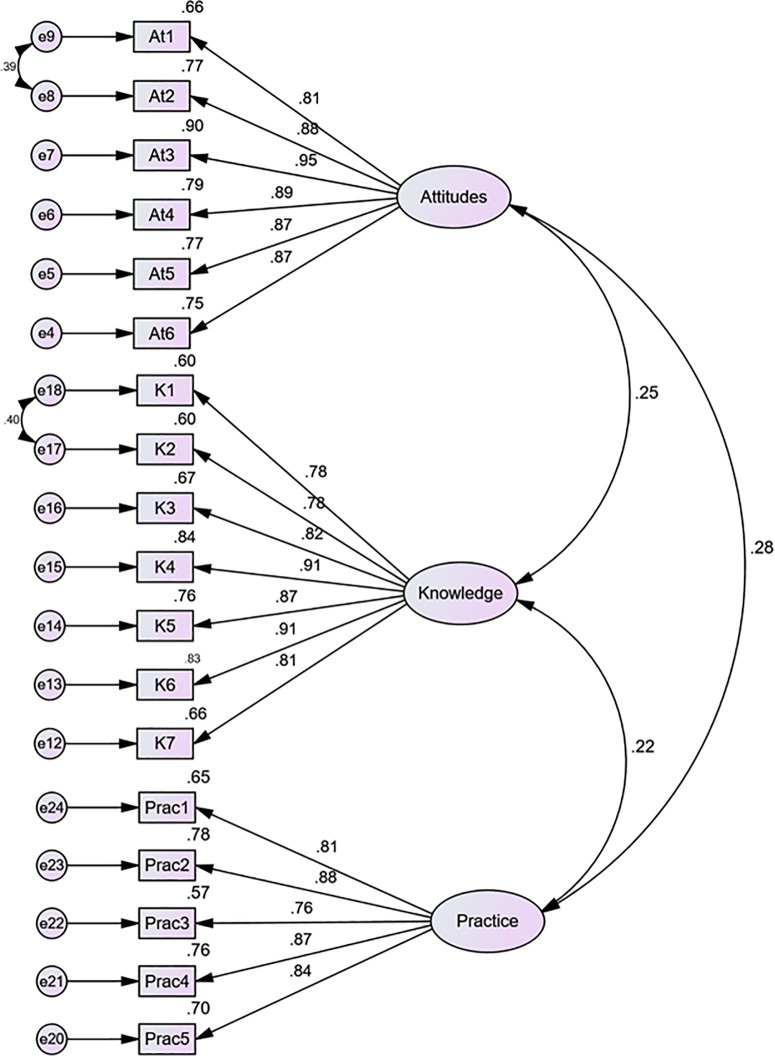
Summary of the confirmatory factor analysis results for the Knowledge, Attitudes, and Practice of AI in Education Scale. *Attitudes* = perceived beliefs and dispositions toward AI in education; *Knowledge* = self-reported understanding of AI tools and concepts; *Practice* = implementation of AI-supported teaching or learning strategies. All standardized factor loadings were statistically significant (*p* < .001).

Confirmatory factor analysis was performed using AMOS to validate the three-factor structure revealed through the EFA (see Fig 2). CFA tested four progressively refined models (Table 3). Model 1 began with the 25 retained items from EFA. Based on low standardized loadings (<.50), two items (K8 and At9) were removed. Model 2 (23 items) showed a marginal improvement. However, Model 3 excluded three additional items (At7, Pract7, and K9) because of the continued weak performance. Model 4, which removed two additional items (At8 and Prac6), resulted in an optimal 18-item solution.

**Table 3:** Summary of model fit indices.

Model Version	df	χ²/df	CFI	TLI	RMSEA	SRMR
Model 1	272	2.471	.883	.871	.096	.0649
Model 2	225	2.503	0.9	0.887	0.097	.0653
Model 3	166	2.413	.921	.910	.094	.0556
Model 4	130	2.105	.947	.937	.083	.0556

Model four yielded excellent fit indices: χ²/df = 2.105, df = 130, p < .001, CFI = .947, TLI = .937, RMSEA = .083 (90% CI [.069,.097]), and SRMR = .0556. While RMSEA (0.083) marginally exceeds 0.08, it remains acceptable, given the sample size and complexity of the model [56]. The final solution included three latent constructs: attitudes toward AI Integration (six items), Knowledge of AI Applications (seven items), and AI-related Pedagogical Practice (five items). All standardized factor loadings ranged from.756 to.949 and were statistically significant (p < .001), indicating strong item loadings and evidence of convergent validity [57].

#### Convergent and discriminant validity.

Convergent and discriminant validity were evaluated using standard criteria from the structural equation modeling literature [57,58]. Convergent validity was supported by strong standardized loadings (.756–.949), high composite reliability values (CR = .918–.953), and acceptable average variance extracted AVE (=.693–.775). Discriminant validity was confirmed using both the Fornell–Larcker criterion and heterotrait-monotrait (HTMT) ratios [58,59]. As shown in Table 4, the square root of each construct’s AVE (bold diagonal) exceeded its interconstruct correlations, satisfying the Fornell–Larcker requirement. Additionally, all the HTMT values were below the conservative threshold of 0.85, providing further evidence of discriminant validity. These findings affirm that the KAP-AIEd instrument possesses robust psychometric properties that align with the theoretical expectations.

**Table 4:** Summary of convergent and discriminant validity of the KAP instrument.

(A) Convergent validity			
**Construct**	**Items (Factor Loadings)**	**CR**	**AVE**
**Attitudes**	At1 (.815), At2 (.877), At3 (.949), At4 (.887), At5 (.875), At6 (.867)	.953	.775
**Knowledge**	K1 (.776), K2 (.776), K3 (.820), K4 (.914), K5 (.870), K6 (.912), K7 (.812)	.945	.774
**Practice**	Prac1 (.805), Prac2 (.882), Prac3 (.756), Prac4 (.872), Prac5 (.840)	.918	.693
**(B) Discriminant validity (Fornell–Larcker Criterion)**			
**Construct**	**Attitudes**	**Knowledge**	**Practice**
**Attitudes**	**0.881**	0.252	0.283
**Knowledge**	0.252	**0.880**	0.222
**Practice**	0.283	0.222	**0.833**

*Note.* CR = composite reliability; AVE = average variance extracted. Values in parentheses are standardized factor loadings. Bolded diagonal values in (B) represent the square root of AVE. The off-diagonal values are inter-construct correlations. All factor loadings were > .70. The HTMT ratios ranged from 0.41 to 0.62, supporting discriminant validity.

#### Reliability.

Internal consistency reliability was evaluated using Cronbach’s alpha. The results indicated excellent reliability across all constructs, with alpha values of 0.954 for *attitude*, 0.944 for *knowledge*, and 0.917 for *practice*. Each coefficient exceeded the commonly accepted threshold of 0.70, confirming a strong internal consistency within the scale [57].

### Procedures

Ethical approval for this study was obtained from the University Research Ethics Committee of Abu Dhabi University (approval no. CAS–000007), in accordance with the Declaration of Helsinki. Prior authorization was also granted by the Department of Education and Knowledge (ADEK) to facilitate data collection in schools across the UAE. Public and private schools across all seven UAE emirates were initially contacted through official email lists and professional educational networks. However, responses were received from four emirates (Abu Dhabi, Dubai, Sharjah, and Ajman). School administrators who agreed to participate distributed the anonymous survey link hosted on Qualtrics to the teaching staff. The survey was conducted in Arabic and English to accommodate language preferences.

An informed consent statement was presented on the first page of the survey, detailing the study’s aims, confidentiality protection, voluntary participation, and data usage. The respondents could proceed only after indicating their consent. Data were collected between December 2023 and March 4, 2024. To reduce non-response bias, three reminder emails were sent at two-week intervals. All responses were anonymous and no identifiable personal or institutional data were collected. Access to the dataset was password-protected and restricted by the research team. Upon completion, the data were stored securely on the university’s encrypted server.

### Data analysis

The dataset was transferred from Microsoft Excel to SPSS, version 28, for cleaning and analysis. The final sample comprised 161 patients. A small proportion of missing data (approximately 2.5%) were identified, primarily in items related to AI-related practices. No cases were excluded, as the extent of missingness was minimal, and data integrity was not compromised.

To assess the pattern of missingness, Little’s Missing Completely at Random (MCAR) test yielded a significant result, χ² [79] = 151.685, *p* < .001, indicating that the data were not missing completely at random. Given this outcome, the data were assumed to be missing at random (MAR) and the expectation-maximization (EM) algorithm was applied to impute missing values. Pre- and post-imputation comparisons revealed negligible differences in means and standard deviations, confirming the stability and robustness of the imputed dataset.

Harman’s single-factor test was conducted to assess the potential for common method bias. The analysis showed that the first unrotated factor accounted for 36.42% of the total variance, well below the 50% threshold, suggesting that the common method variance was not a significant concern [60].

To address RQ1, descriptive statistics including means and standard deviations were computed for the three latent constructs. Composite mean scores were calculated by averaging the relevant items within each construct. In line with interpretive conventions for Likert-type data, mean values approaching 4.00 were considered indicative of generally positive perceptions, awareness, or implementation of AI in education [61].

To address RQ2, a factorial multivariate analysis of variance (MANOVA) was conducted to examine whether teachers’ attitudes, knowledge, and AI-related practices differed significantly across demographic variables. Prior to analysis, assumptions of multivariate normality, homogeneity of variance–covariance matrices, and absence of multicollinearity were assessed and met. Wilks’ Lambda (Λ) was used as the multivariate test statistic. For the demographic variables with significant multivariate effects, follow-up univariate ANOVAs were performed for each dependent variable. Where appropriate, post hoc comparisons were conducted using Tukey’s HSD. A Bonferroni-adjusted alpha level (α = .0125) was applied to control for Type I error inflation in multiple comparisons [55]. Partial eta squared (η²) was reported as a measure of effect size and interpreted using guidelines [62]: small (η² ≥ .01), medium (η² ≥ .06), and large (η² ≥ .14).

To address RQ3, structural equation modeling (SEM) was conducted using AMOS 26.0. SEM was selected for its capacity to model direct and indirect predictive pathways among latent constructs, while accounting for measurement error [63]. The hypothesized model examined whether teachers’ attitudes and knowledge predicted AI-related pedagogical practices consistent with the KAP framework. Model fit was evaluated using widely accepted criteria: chi-square to degrees of freedom ratio (χ²/df) < 5.00, Comparative Fit Index (CFI), Tucker-Lewis Index (TLI) ≥ 0.92, Root Mean Square Error of Approximation (RMSEA), and Standardized Root Mean Square Residual (SRMR) ≤ 0.08 [64,65]. The amount of variance explained (R²) was assessed to evaluate the predictive power of the model.

In addition to testing direct effects, bootstrapped mediation analysis with 5,000 resamples was conducted to evaluate the indirect effect of knowledge on practice via attitudes. Bias-corrected 95% confidence intervals were used to assess the statistical significance of the mediation effect, following best practices in SEM mediation testing.

To address Research Question 4, whether demographic variables moderate the relationship between teachers’ knowledge or attitudes and their AI-related practices, moderation analyses were conducted using Hayes’ PROCESS macro (Model 1, version 4.2) in SPSS [66]. Separate moderation models were specified for each demographic variable and predictor (knowledge or attitude). Interaction terms were computed as the product of the mean-centered predictor and moderator variables. All analyses used bootstrapping with 5,000 resamples and 95% bias-corrected confidence intervals to enhance the estimation robustness. The statistical significance for the interaction effects was evaluated at α = .05.

## Results

### RQ1: Perceived Levels of Knowledge, Attitudes, and Practice Toward AI

Descriptive statistics were computed to summarize teachers’ self-reported attitudes toward AI, knowledge of AI applications, and AI-related instructional practices. Participants reported high attitudes (M = 3.95, SD = 0.83) and moderate knowledge (M = 3.81, SD = 0.91), whereas their reported classroom practice of AI was comparatively lower (M = 3.17, SD = 1.10).

The item-level means are summarized in Table 5. Among attitude items, the highest-rated statements were “People should learn AI technology for the future needs of the education sector” (At1, M = 4.11, SD = 0.80) and “I am willing to use AI technology for developing smart content” (At2, M = 4.07, SD = 0.88). The knowledge items that received the highest agreement included awareness of AI’s limitations in education (K6, M = 3.99, SD = 1.05) and familiarity with AI’s general applications (K1, M = 3.90, SD = 1.12).

**Table 5:** Summary of item-level means and standard deviations for the KAP instrument.

Item code	Item Description	M	SD
**Attitudes Toward AI in Education**			
At1	People should learn AI technology to meet future educational needs	4.11	0.803
At2	I am willing to use AI technology to develop smart content	4.07	0.877
At3	I would recommend stakeholders explore AI technology for academic use	3.93	0.956
At4	AI applications in education can make learning more interactive	3.94	0.957
At5	AI can help make education more cost-effective	3.74	1.022
At6	AI can make teaching and learning more engaging	3.89	0.880
**Knowledge of AI Applications**			
K1	I use AI tools because they make learning more impactful and engaging	3.90	1.119
K2	I am familiar with AI applications in the education sector	3.81	1.121
K3	I understand the concept of big data and its relationship to AI	3.76	1.106
K4	I am aware of the ethical and social implications of AI	3.83	0.985
K5	I am familiar with AI applications in fields like healthcare, finance, or transport	3.68	0.945
K6	I am aware that AI has limitations in teaching and learning	3.99	1.052
K7	I understand the role of logical reasoning and causal analysis in AI applications	3.67	1.059
**Practice of AI in Teaching and Learning**			
Prac1	I conduct assessments using AI tools	3.09	1.312
Prac2	I use AI to enhance student engagement	3.28	1.174
Prac3	I plan academic support based on AI-generated insights	3.10	1.305
Prac4	I use AI-powered adaptive learning materials	3.16	1.219
Prac5	I design learning outcomes using AI-guided tools	3.23	1.343

Within the practice domain, teachers most frequently reported using AI to enhance student engagement (Prac2, M = 3.28, SD = 1.17) and designing learning outcomes using AI-guided tools (Prac5, M = 3.23, SD = 1.34). The lowest-rated item was conducting assessments using AI tools (Prac1, M = 3.09, SD = 1.31).

### RQ2: Group Differences in Attitudes, Knowledge, and Practice Toward AI

A MANOVA was conducted to examine whether teachers’ demographic characteristics were associated with differences in their reported attitudes, knowledge, and AI-related practices. Table 6 presents the results of the multivariate tests and follow-up univariate ANOVAs.

**Table 6:** Differences in attitudes, knowledge, and practice toward AI by demographic variables.

Variable	Wilks’ Λ	MANOVA F (df)	Attitudes F (η²)	Knowledge F (η²)	Practice F (η²)
Gender	0.950	2.647* (3, 157)	5.581* (0.034)	0.203 (0.001)	1.184 (0.007)
School Type	0.863	7.921** (3, 157)	23.791** (0.132)	50.986** (0.245)	13.091** (0.077)
Gender × School Type	0.901	3.884** (3, 157)	21.537** (0.121)	2.618 (0.016)	7.848** (0.048)
Teaching Experience	0.897	1.594* (9, 153)	3.270* (0.070)	0.432 (0.010)	0.874 (0.020)
Age	0.831	2.069* (12, 152)	2.980* (0.083)	0.914 (0.027)	1.501 (0.044)
Academic Position	0.861	2.663** (9, 153)	1.378 (0.026)	4.845** (0.085)	3.747* (0.067)

*Note.* Wilks’ Λ = Wilks’ Lambda; η² = partial eta squared. Asterisks denote significance levels: *p* < .05 = *, *p* < .01 = **. The MANOVA F-statistics are reported with degrees of freedom in parentheses. The non-significant values are unmarked.

A significant multivariate effect was observed for school type (Wilks’ Λ = 0.863, F(3, 157) = 7.921, p < .001, η² = .245. Follow-up univariate analyses indicated that teachers in private schools scored significantly higher than those in public schools on attitude (F(1, 157) = 23.791, p < .001, η² = .132), knowledge (F(1, 157) = 50.986, p < .001, η² = .245), and practice (F(1, 157) = 13.091, p < .001, η² = .077).

Gender also showed a significant multivariate effect (Wilks’ Λ = 0.950, F ([3], 157) = 2.647, p = .046, η ² = .050). The univariate results revealed that female teachers reported significantly more positive attitudes toward AI than male teachers (F(1, 157) = 5.581, p = .019, η² = .034), although no significant differences were found for knowledge or practice.

A significant interaction between gender and school type was detected (Wilks’ Λ = 0.901, F(3, 157) = 3.884, p = .006, η² = .121. A simple effects analysis showed that female teachers in private schools reported the highest levels of positive attitudes (M = 4.5, SD = 0.8). Significant interaction effects were also found for attitude (F(1, 157) = 21.537, p < .001, η² = .121) and practice (F(1, 157) = 7.848, p = .006, η² = .048).

A significant multivariate effect was also observed for teaching experience (Wilks’ Λ = 0.897, F ([9], 153) = 1.594, p = .035, η ² = .036). Post-hoc Bonferroni-adjusted univariate analyses showed significant differences in attitudes (F(3, 157) = 3.270, p = .023, η² = .070). Teachers with 4–6 years of experience (M = 4.2) scored significantly higher than those with 1–3 years (M = 3.5, p = .008) and 7–10 years (M = 3.6, p = .003). Teachers with more than 10 years of experience (M = 4.0) also scored significantly higher than those with 7–10 years of experience (p = .040).

A significant multivariate effect was also found for age (Wilks’ Λ = 0.831, F ([12], 152) = 2.069, p = .016, η ² = .060). Univariate analyses showed significant differences in attitudes (F(4, 157) = 2.980, p = .022, η² = .083), with teachers aged 51–60 years (M = 4.3) scoring significantly higher than those aged over 60 years (M = 3.7, p = .027).

Lastly, academic position was associated with significant group differences (Wilks’ Λ = 0.861, F ([9], 153) = 2.663, p = .005, η ² = .049). Univariate analyses revealed significant differences in knowledge (F(1, 157) = 4.845, p = .029, η² = .085), with General Education Teachers (M = 4.1, SD = 0.6) scoring significantly higher than Special Education Teachers (M = 3.4, SD = 0.9, p = .014). Similarly, significant differences were observed in practice (F(1, 157) = 3.747, p = .028, η² = .067), where General Education Teachers (M = 3.8, SD = 0.8) outperformed Special Education Teachers (M = 3.1, SD = 0.7, p = .014).

### RQ3: Predicting AI-related practice through path analysis

Structural equation modeling was conducted to examine whether teachers’ attitudes and knowledge significantly predicted their AI-related pedagogical practices. The hypothesized model yielded acceptable fit indices, χ²/df = 2.105, RMSEA = .083, CFI = .947, TLI = .937, and SRMR = .051, meeting the widely accepted criteria for model adequacy.

**Fig 3:**
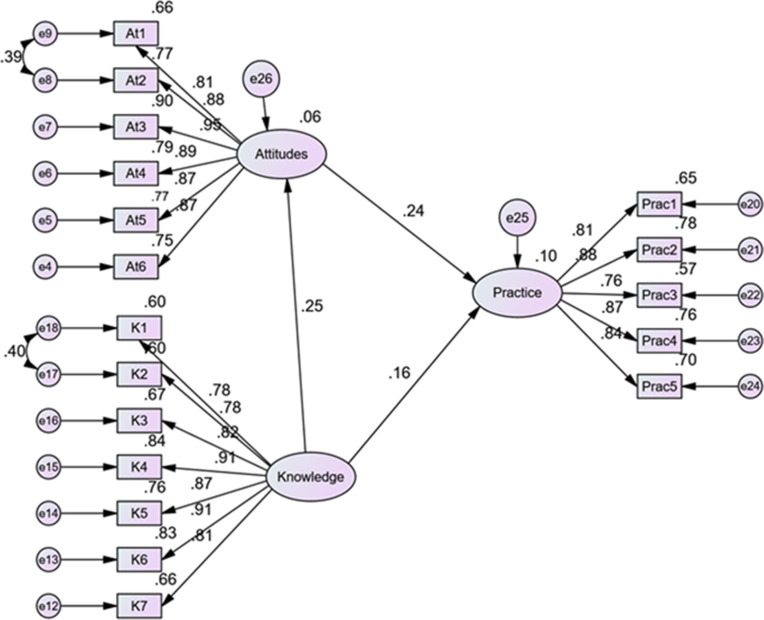
Final structural equation model showing standardized path coefficients. Attitudes significantly predicted AI-related practice (β = .322, p = .004), while the path from Knowledge to Practice was marginally non-significant (β = .200, p = .056). A bootstrapped analysis confirmed a significant indirect effect of Knowledge on Practice via Attitudes (β = .076, p = .006, 95% CI: [.072,.528]). All factor loadings are significant (p < .001).

As shown in Fig 3, attitudes significantly predicted AI-related practices (β = .322, *p* = .004), indicating that more positive attitudes were associated with greater implementation of AI in instructional settings. The direct path from knowledge to practice was positive but marginally non-significant (β = .200, *p* = .056). The model explained 10.4% of the variance in AI-related practices (R² = .104), with attitudes demonstrating a stronger standardized effect.

To assess the indirect relationship hypothesized in the conceptual framework, we conducted bootstrapped mediation analysis with 5,000 samples. The results indicated that knowledge had a significant indirect effect on AI-related practices through attitudes (β = .076, p = .006), with a bias-corrected 95% confidence interval of [.072,.528]. This supports the mediation pathway specified in the KAP model and confirms that attitudes partially mediate the effect of knowledge on practice.

These findings partially support the hypothesized model. Hypothesis 2, which proposes a significant positive relationship between attitudes and practices, is supported. Although the direct effect of knowledge on practice was only marginally significant, the significant indirect effect supported hypothesis 1 in the context of partial mediation.

#### Latent correlations among constructs.

Latent correlations derived from the CFA model further supported the theoretical framework (Fig 2). Attitudes were positively correlated with knowledge (r = .25, p = .004) and practice (r = .28, p < .001), while knowledge showed a significant positive correlation with practice (r = .22, p < .01). These associations reinforce the internal coherence of the model and directional logic of the hypothesized pathways.

### RQ4: Moderation effects of demographic variables on the relationships between knowledge, attitudes, and ai practice

**Fig 4:**
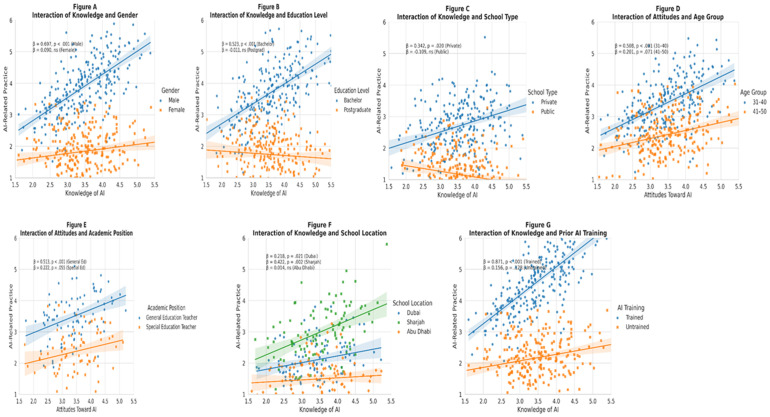
Interaction effects of key demographic moderators on the relationship between AI knowledge or attitudes and AI-related practice. *Note.* Panels A–G illustrate significant moderation effects across demographic groups. Each plot shows simple slopes with 95% confidence intervals. Moderators include Gender **(A)**, Education Level **(B)**, School Type **(C)**, Age Group **(D)**, Academic Position **(E)**, School Location **(F)**, and Prior AI Training **(G)**. These figures demonstrate how demographic variables significantly influenced the strength or direction of the relationship between AI-related Knowledge or Attitudes and Practice. Full regression statistics are reported in Table 7.

A series of moderation analyses was conducted to examine whether selected demographic characteristics moderated the effects of AI-related knowledge and attitudes on teachers’ reported AI-related practices. Several significant interaction effects emerged, as summarized in Table 7 and visualized in Fig 4 (Panels A–G).

**Table 7:** Summary of moderation analysis results: Interaction and conditional effects.

Moderator	Effect Type	Term/ Subgroup	β	SE	t	p	95% CI	Sig.
**Gender**	Interaction	Knowledge × Gender	−0.607	0.225	−2.70	0.008	[-1.051, -0.163]	**
	Conditional	→ Male	0.697	0.199	—	0.0006	[0.304, 1.090]	**
		→ Female	0.090	0.104	—	0.388	[-0.115, 0.295]	ns
**Education level**	Interaction	Knowledge × Education Level	−0.267	0.093	−2.88	0.005	[-0.449, -0.084]	**
	Conditional	→ Bachelor’s	0.523	0.140	—	0.0003	[0.247, 0.799]	**
		→ Postgraduate	−0.011	0.121	—	0.928	[-0.250, 0.228]	ns
**School type**	Interaction	Knowledge × School Type	−0.451	0.211	−2.14	0.034	[-0.867, -0.036]	*
	Conditional	→ Private schools	0.342	0.146	—	0.020	[0.054, 0.630]	*
		→ Public schools	−0.109	0.152	—	0.473	[-0.409, 0.191]	ns
**Age**	Interaction	Attitudes × Age	−0.307	0.108	−2.84	0.005	[-0.520, -0.093]	**
	Conditional	→ 31–40 years	0.508	0.117	—	0.000	[0.277, 0.739]	**
		→ 41–50 years	0.201	0.111	—	0.071	[-0.018, 0.420]	^†^
**Academic position**	Interaction	Attitudes × Academic Position	−0.270	0.130	−2.08	0.039	[-0.527, -0.013]	*
	Conditional	→ General education teacher	0.513	0.133	—	0.0002	[0.251, 0.775]	**
		→ Special education teacher	0.222	0.115	—	0.055	[-0.005, 0.449]	^†^
**School location**	Interaction	Knowledge × School Location	0.204	0.100	2.03	0.044	[0.006, 0.402]	*
	Conditional	→ Dubai	0.218	0.093	—	0.021	[0.034, 0.402]	*
		→ Sharjah	0.422	0.134	—	0.002	[0.157, 0.687]	**
		→ Abu Dhabi	0.014	0.140	—	0.922	[-0.262, 0.290]	ns
**Prior AI training**	Interaction	Knowledge × Prior AI Training	0.715	0.246	2.91	0.004	[0.229, 1.202]	**
	Conditional	→ Trained	0.871	0.224	—	0.0001	[0.428, 1.314]	**
		→ Untrained	0.156	0.102	—	0.128	[-0.044, 0.356]	ns

*Note.* This table summarizes the significant and non-significant moderating effects of demographic variables on the relationships between AI knowledge or attitudes and AI-related practices. β = unstandardized coefficient; SE = standard error; CI = confidence interval. Significance: **p** < .01 = **, *p* < .05 = *,*

† *p* < .10 = marginal, ns = not significant.

A significant interaction was observed between Knowledge and Gender in predicting AI-related practices (β = −0.607, SE = 0.225, p = .008, 95% CI [−1.051, −0.163]). Conditional effects analysis revealed that knowledge significantly predicted practice among male teachers (β = 0.697, p < .001) but not among female teachers (β = 0.090, p = .388; see Fig 4A).

Education Level also significantly moderated the knowledge-practice relationship (β = −0.267, SE = 0.093, p = .005). Knowledge was a significant predictor of practice for teachers with a bachelor’s degree (β = 0.523, p < .001) but not for those with postgraduate qualifications (β = −0.011, p = .928; Fig 4B).

A significant interaction was found between Knowledge and School Type (β = −0.451, SE = 0.211, p = .034). Knowledge predicted significantly higher practice among private schoolteachers (β = 0.342, p = .020), but the relationship was not significant among public schoolteachers (β = −0.109, p = .473; Fig 4C).

With regard to age, a significant interaction was detected between Attitudes and Age groups (β = −0.307, SE = 0.108, p = .005). Attitudes predicted AI-related practices most strongly among teachers aged 31–40 years (β = 0.508, p < .001), while the effect was weaker and marginal among those aged 41–50 years (β = 0.201, p = .071; Fig 4D).

Academic Position also moderated the attitude–practice relationship (β = −0.270, SE = 0.130, p = .039). Attitudes significantly predicted Practice for General Education Teachers (β = 0.513, p < .001), but the effect was only marginal for Special Education Teachers (β = 0.222, p = .055; Fig 4E).

A significant interaction was observed between Knowledge and School Location (β = 0.204, SE = 0.100, p = .044). Knowledge significantly predicted AI-related practices in Dubai (β = 0.218, p = .021) and Sharjah (β = 0.422, p = .002) but not in Abu Dhabi (β = 0.014, p = .922; Fig 4F).

Finally, Prior AI Training significantly moderated the knowledge–practice relationship (β = 0.715, SE = 0.246, p = .004). Among teachers who had received AI training, knowledge strongly predicted practice (β = 0.871, p < .001), while the effect was non-significant among those without training (β = 0.156, p = .128; Fig 4G).

No significant interaction effects were found for Teaching Experience, Knowledge × Age, Knowledge × Academic Position, Attitudes × School Location, or Attitudes × AI Training, as shown in Table 7.

## Discussion

This study investigated the levels and relationships among teachers’ knowledge, attitudes, and AI-related practices in UAE K–12 schools, as well as the moderating role of demographic characteristics. Overall, the findings reveal a pattern of strong attitudes and moderate knowledge, in contrast with lower levels of AI implementation in classroom practice. Additionally, both the structural and moderating effects underscore the importance of individual and institutional factors in shaping AI readiness. The following sections interpret these findings in light of existing literature and theory, highlighting their implications for professional development, policy, and future research.

### Teachers’ knowledge, attitudes, and practices toward AI

The findings revealed that the study participants reported relatively high levels of AI-related attitudes and moderate knowledge, with AI-related classroom practices rated slightly lower overall. While the composite mean for practice was modest, item-level analysis revealed more nuanced patterns; for example, using AI for student engagement and designing learning outcomes was reported more frequently, while conducting assessments using AI tools was less common. These findings suggest selective and context-dependent implementation rather than uniformly low practice levels.

This emerging pattern aligns with previous research showing that positive attitudes and general awareness often precede consistent integration into pedagogical routines [10,16,41]. Rather than reflecting a sharp readiness–implementation gap, the data indicate that teachers begin to experiment with specific AI applications while still navigating systemic constraints.

Several factors may have accounted for this discrepancy. In their study, Gayed (2025) suggested that educators often possess fragmented or superficial AI knowledge, which hinders confidence and sustained usage in teaching. Moreover, institutional constraints, including limited infrastructure, a lack of curricular alignment, and insufficient training opportunities, have been documented in both MENA [11] and global studies [35,67]. These barriers highlight the importance of well-designed professional development programs that address both technical competence and pedagogical integration [19,68].

Overall, the results underscore the need for capacity-building strategies that go beyond awareness to foster a deeper pedagogical appropriation of AI. Bridging the gap between perception and practice requires equipping teachers with tools, training, and confidence to meaningfully apply AI in diverse instructional contexts.

### Group differences in knowledge, attitudes, and practice

Significant differences in teachers’ knowledge, attitudes, and practices regarding AI were observed across several demographic categories. Consistent with prior UAE-based studies [10,11], teachers in private schools reported higher levels across all three KAP domains than their public-school counterparts. These findings likely reflect a more innovation-oriented culture, greater institutional autonomy, and increased access to technology typically associated with private schools. Prior research underscores the importance of organizational support and resource availability in shaping AI readiness [35,67].

Gender differences also emerged, with female teachers reporting more favorable attitudes toward AI. This finding aligns with prior research suggesting that women in education may perceive greater pedagogical value in AI applications or respond more positively to professional development opportunities related to technology [33,69]. However, this attitudinal advantage does not consistently translate into higher levels of AI practice, echoing international studies emphasizing the complexity of gendered technology adoption patterns [25].

Contrary to the assumption that early career or younger teachers would show higher AI readiness, mid-career educators (with 4–6 years of experience) and teachers aged 51–60 reported significantly more positive attitudes toward AI. These results diverge from prior research emphasizing youth-driven digital enthusiasm [24] and suggest that digital maturity may not be strictly age bound. Instead, growing professional expectations and system-wide initiatives around digital transformation may prompt greater openness among mid- and late-career teachers [20].

Academic role also influences KAP outcomes. General Education Teachers scored significantly higher than Special Education Teachers in both knowledge and AI-related practice. This result supports prior research indicating that special education professionals often face additional barriers to adopting emerging technologies [13,31]. These challenges may stem from the lack of AI tools adapted to the diverse needs of students with disabilities as well as limited access to specialized training that integrates inclusive pedagogy with AI-driven instructional practices.

Taken together, these demographic disparities underscore the need for differentiated equity-conscious professional development strategies. Ensuring that AI integration reaches all educators across school types, roles, genders, and career stages requires policy attention to systemic factors that enable or inhibit adoption. While these group differences offer valuable insights, it is important to recognize that the sample was concentrated in private schools and urban emirates, where resource access and institutional flexibility may be more prevalent. As such, the observed differences may reflect contextual advantages that are not equally available in public or rural school settings. This limits the generalizability of the findings and suggests that public school teachers in less-resourced areas may face distinct challenges when adopting AI. Future research should explore how governance structures, infrastructure, and the geographic context interact with individual readiness to inform more equitable AI implementation strategies.

### Predicting AI practice: role of attitudes and knowledge

This study found that teachers’ attitudes played a stronger role than their knowledge in predicting their use of AI in instructional settings. While both constructs were positively associated with practice, only attitudes emerged as a statistically significant predictor, suggesting that favorable beliefs about AI’s pedagogical value may be more influential than technical understanding alone.

Importantly, bootstrapped mediation analysis revealed a significant indirect effect of knowledge on practice through attitudes, providing empirical support for the mediation pathway specified in the conceptual framework. This confirms that attitudes partially mediate the relationship between knowledge and practice, which is consistent with the KAP model. According to the KAP theory, knowledge enhances behavior only when it is supported by favorable attitudes and enabling conditions.

These findings also align with TAM, which posits that attitudes and perceived usefulness are critical determinants of technology adoption [32]. Teachers who viewed AI as beneficial for engagement, personalization, and instructional innovation were more likely to integrate it into their instruction, even when their conceptual knowledge was limited.

Moreover, the findings reinforce the core principles of the KAP model, which emphasize that knowledge does not automatically translate into action unless accompanied by positive affect and contextual support. This is consistent with recent research on AI-integrated education, which has found that knowledge alone is insufficient without motivation, confidence, or institutional encouragement [8,20,69].

Although the direct path from knowledge to practice was marginally nonsignificant (p = .056), its proximity to the conventional threshold suggests that knowledge may still exert a meaningful influence under certain conditions. This interpretation was strengthened by the moderation analysis, which revealed that knowledge significantly predicted AI-related practices among teachers who had received prior AI training. These findings imply that knowledge becomes behaviorally relevant when accompanied by structured experiential support, highlighting the importance of contextual factors such as training and implementation opportunities.

The marginal effect of knowledge may reflect broader challenges in teacher preparation as surface-level exposure to AI concepts does not automatically result in actionable pedagogical competence. Professional development must go beyond awareness to build confidence, pedagogical framing, and relevance [25]. In this light, promoting AI adoption requires not only cognitive input, but also emotional and practical scaffolding.

Overall, the results suggest that capacity-building efforts should foreground attitudinal development and perceived pedagogical value rather than focus solely on technical literacy. Helping teachers answer the question “Why should I use AI?” may be as important if not more so than “How does AI work?”

### Moderation effects

The moderation analysis revealed that several demographic variables shaped how teachers’ knowledge and attitudes translated into AI-related practices. These findings offer important insights into which subgroups of teachers are most likely to benefit from AI-related training and capacity-building efforts, which may require tailored support.

Gender emerged as a key moderator in the knowledge–practice relationship. Although female teachers reported more favorable attitudes overall, knowledge significantly predicted practice only among male teachers. This suggests that, while attitudinal support is higher among females, cognitive familiarity with AI has a greater behavioral impact among males. These findings align with those of earlier studies [33,69], highlighting the nuanced gender dynamics in technology adoption, in which social encouragement and perceived ease of use interact differently across male and female educators.

Education level also moderated the knowledge–practice pathway, with significant effects observed among teachers holding bachelor’s degrees but not among those with postgraduate qualifications. The observed moderating effect suggests that the relationship between AI knowledge and practice differs according to educational attainment. Teachers with a bachelor’s degree demonstrated a significant knowledge practice link, whereas those with postgraduate qualifications did not. While this could reflect divergent pedagogical approaches or role expectations (e.g., postgraduate educators often have administrative duties that reduce classroom technology implementation), our data cannot definitively explain this disparity. These findings are supported by [70], who found that digital competence and openness to AI use were not necessarily higher among academically qualified teachers. Similarly, [67] emphasized that professional development, not educational level, was the strongest predictor of AI integration in classroom practice.

The school type continued to play a crucial role. Knowledge is a significant predictor of AI practice in private schools, but not in public ones, reinforcing earlier results and echoing global findings that institutional flexibility, access to resources, and leadership support are critical enablers of innovation [35,67].

Similarly, age and academic position moderated the effect of attitude on practice. Teachers aged 31–40 years showed the strongest link between attitudes and AI use, suggesting mid-career openness to innovation. General Education Teachers, in contrast to their Special Education counterparts, are more likely to act on their positive perceptions of AI, possibly because of differences in classroom demands, training exposure, or the limited availability of inclusive AI tools [13,31].

Crucially, prior AI training enhanced the knowledge–practice relationship. Among trained teachers, knowledge strongly predicted AI use, while no such effect was observed in the untrained group. This finding emphasizes the importance of hands-on, relevant professional development, echoing calls for AI-focused training that bridges conceptual understanding with practical classroom applications [67,68,70].

Finally, school location influenced the knowledge–practice link, with stronger effects observed in Dubai and Sharjah than in Abu Dhabi. This suggests that contextual factors, such as access to infrastructure, local policy priorities, or networking opportunities, may shape how readily teachers can act on their AI knowledge. These geographic disparities highlight the need for equity-driven location-sensitive AI integration strategies across the UAE.

## Implications

### Implications for policy

These findings underscore the need for systemic strategies that support equitable and effective AI integration in education, both within the UAE and in comparable international contexts. Nationally, disparities in teacher readiness by school type, academic role, and geographic location highlight the urgency of targeted investment in digital infrastructure, particularly in public and rural schools. Policymakers should be encouraged to develop differentiated AI readiness frameworks that account for varying levels of teacher expertise, prior training, and access to institutional resources.

On a global scale, the UAE serves as a case study on how national AI strategies can facilitate school-level transformation when aligned with inclusive teacher development. Countries seeking to implement or scale AI in education should prioritize digital equity, comprehensive professional development, and incentives for innovation. This study contributes to international discourse by illustrating how structural conditions such as governance, resource distribution, and support systems can shape the success of national AI reforms.

### Implications for practice

At the practitioner level, the findings emphasize the importance of differentiated and inclusive professional development. Teachers with positive attitudes toward AI were more likely to report implementation, even in the absence of advanced technical knowledge. As such, professional development programs should focus not only on skill building, but also on cultivating positive dispositions toward AI’s pedagogical value. Reflective hands-on training tailored to diverse educator profiles, including special education teachers and those with postgraduate qualifications, will be critical for bridging the gap between readiness and practice.

School leadership also plays a vital role in fostering a culture of innovation. Initiatives that promote peer collaboration, provide time for experimentation, and incorporate AI into instructional standards collectively support sustainable adoption. This study suggests that school-based interventions should accommodate varying levels of teacher experience and regional contexts to ensure equitable access to the benefits of AI in education.

### Theoretical implications

The results extend and reinforce two key conceptual frameworks: the KAP model and the TAM. Consistent with these theories, attitudes have emerged as a stronger predictor of AI-related practices than knowledge, highlighting the motivational and affective dimensions of technology adoption. Furthermore, the significant moderating effects of the demographic variables suggest that both models should be applied with sensitivity to contextual and institutional differences. This study contributes to theoretical refinement by demonstrating how teacher-level characteristics and school environments interact to influence the transition from beliefs to classroom behaviors. Future research applying the KAP and TAM in education should incorporate differentiated learning environments and role-specific demands to better capture the complexity of innovation adoption across diverse teaching populations.

### Limitations and future research

While this study provides valuable insights into UAE K–12 teachers’ readiness for AI integration, several limitations should be acknowledged. First, the cross-sectional design precludes causal inferences among the core constructs. Although structural equation modeling identifies directional relationships, longitudinal or intervention-based studies are needed to assess how teacher readiness evolves over time in response to training or policy shifts.

Second, the use of self-reported survey data introduces the possibility of social desirability bias, especially in relation to the reported practices. Despite the anonymity of the survey, participants may have overestimated their engagement with AI tools. Future studies could benefit from triangulating survey data with classroom observations, usage logs, or lesson materials to validate the implementation.

Third, a major limitation of this study is the under-representation of public school teachers (21%) and the geographic concentration of the sample in only four of the seven Emirates, primarily in urban areas. This sample imbalance, combined with the predominance of private-sector teachers, may limit the external validity of the findings. Given that the MANOVA analyses revealed significant differences across all KAP domains between public and private schoolteachers, generalizing these results to the broader UAE teaching population should be approached with caution. The findings are best interpreted as reflective of trends among general and special education teachers working in sampled urban, private, and public school settings, rather than as representative of all teachers nationwide.

Finally, the study did not account for systemic variables, such as leadership support, curriculum autonomy, or technological infrastructure, which may significantly influence teachers’ capacity to adopt AI. Future investigations should consider multilevel or mixed-methods approaches to explore how institutional and contextual factors interact with teacher-level readiness. Additionally, research on the impact of differentiated professional development, particularly for underrepresented groups, such as special educators, would deepen our understanding of equitable AI implementation. Exploring student perspectives and learning outcomes in relation to AI use by teachers also offers an important avenue for future research.

Furthermore, the observed moderation effect of educational attainment, where knowledge predicted practice only for teachers with a bachelor’s degree, raises important questions. Future research should examine why postgraduate training appears to weaken the knowledge–practice link, whether due to curricular gaps, confidence barriers, or role-related factors. Mixed-methods studies are needed to understand how advanced academic training shapes teachers’ technology adoption behaviors in practice-focused educational contexts.

## Conclusion

This study examined general and special education teachers’ knowledge, attitudes, and AI-related practices in selecting public and private K–12 schools across four emirates of the UAE. The findings offer timely insights into how demographic and contextual factors shape educators’ readiness for AI integration, particularly in urban resource-accessible school environments.

While attitudes toward AI were generally positive and knowledge levels moderately high, actual classroom implementation remained limited. Structural equation modeling confirmed that attitudes were the strongest direct predictors of AI-related pedagogical practices. Additionally, bootstrapped mediation analysis revealed a significant indirect effect of knowledge on practice through attitudes, highlighting that technical understanding alone is insufficient without attitudinal support. These findings affirm the theoretical value of the KAP model and emphasize the importance of both the cognitive and motivational components in driving AI adoption.

Significant subgroup differences by gender, academic role, and prior AI training underscore the need for differentiated, equity-conscious capacity-building strategies. By integrating the KAP framework with TAM, this study contributes both theoretically and empirically to the emerging discourse on AI in education.

However, these findings should be interpreted cautiously. The sample was disproportionately drawn from private schools (79%) and limited to four of the seven emirates, primarily in urban centers, such as Abu Dhabi and Dubai. Given this concentration and the significant differences observed between public and private schoolteachers across KAP domains, the results should not be generalized to the national teacher population. Instead, they reflect readiness trends within a subset of educators operating in relatively well-resourced innovation-oriented school contexts.

In light of the UAE’s national AI strategy and investments in smart learning environments, this study highlights the urgent need for inclusive policy-aligned professional development. Preparing educators for responsible AI integration requires not only technical training, but also structural and emotional support, especially in public and underserved school contexts. Future research should further investigate the unique challenges faced by public sector teachers and those in rural areas to inform equitable AI implementation across the full spectrum of the UAE’s education system.
